# Acceleration of Polybutylene Succinate Biodegradation by *Terribacillus* sp. JY49 Isolated from a Marine Environment

**DOI:** 10.3390/polym14193978

**Published:** 2022-09-23

**Authors:** Su Hyun Kim, Jang Yeon Cho, Do Hyun Cho, Hee Ju Jung, Byung Chan Kim, Shashi Kant Bhatia, See-Hyoung Park, Kyungmoon Park, Yung-Hun Yang

**Affiliations:** 1 Department of Biological Engineering, College of Engineering, Konkuk University, Seoul 05029, Korea; 2 Institute for Ubiquitous Information Technology and Applications, Konkuk University, Seoul 05029, Korea; 3 Department of Biological and Chemical Engineering, Hongik University, Sejong 30016, Korea

**Keywords:** polybutylene succinate (PBS), *Terribacillus goriensis*, biodegradation

## Abstract

Polybutylene succinate (PBS) is a bioplastic substitute for synthetic plastics that are made from petroleum-based products such as polyethylene and polypropylene. However, the biodegradation rate of PBS is still low and similar to that of polylactic acid (PLA). Moreover, our knowledge about degrader species is limited to a few fungi and mixed consortia. Here, to identify a bacterial degrader to accelerate PBS degradation, we screened and isolated *Terribacillus* sp. JY49, which showed significant degradability. In order to optimize solid and liquid culture conditions, the effect of factors such as temperature, additional carbon sources, and salt concentrations on degradation was confirmed. We observed a degradation yield of 22.3% after 7 days when adding 1% of glucose. Additionally, NaCl was added to liquid media, and degradation yield was decreased but PBS films were broken into pieces. Comparing the degree of PBS degradation during 10 days, the degradation yield was 31.4% after 10 days at 30 °C. Alteration of physical properties of films was analyzed by using scanning electron microscopy (SEM), gel permeation chromatography (GPC), and Fourier transform infrared (FT-IR). In addition, *Terribacillus* sp. JY49 showed clear zones on poly(butylene adipate-co-terephthalate) (PBAT), polycaprolactone (PCL), and copolymers such as P(3HB-*co*-3HV) and P(3HV-*co*-4HB), exhibiting a broad spectrum of degradation activities on bioplastics. However, there was no significant difference in absorbance when esterase activity was examined for different types of bioplastics. Overall, *Terribacillus* sp. JY49 is a potential bacterial strain that can degrade PBS and other bioplastics, and this is the first report of *Terribacillus* sp. as a bioplastic degrader.

## 1. Introduction

Pollution in the oceans is a serious issue that is rapidly increasing due to the waste discharged into water bodies. Increasing marine waste, water pollution, and the formation of garbage islands in the ocean threaten the habitats of marine organisms [1,2]. Synthetic plastics greatly contribute to pollution because of their high usage and improper recycling. Synthetic plastics are artificially produced from petroleum oil with long chains of monomers [3,4]. They are inexpensive and easy to process. Therefore, they are found in manufacturing and packaging goods and pharmaceutical industries [5,6,7,8]. Because of their low cost and lightweight nature, we use them frequently in our day-to-day lives [9,10]. Moreover, their chemical structure allows their easy manipulation into shapes, makes them non-reactive, and increases their durability. However, their inert nature and durability make them hard to decompose in nature [11,12], rendering them difficult to dispose of, thus leading to serious environmental problems. 

Therefore, the use of biodegradable bioplastics made from bio-based materials is increasing. Bio-based bioplastics are produced from biomass such as sugarcane, cellulose, or corn instead of fossil fuel. They can even be produced from microbes under various culture conditions [13]. Biodegradable bioplastics are converted to natural substances such as water and carbon dioxide by microorganisms [14]. Therefore, they are gaining attraction as substitutes for petroleum-based plastics. There are various kinds of bioplastics: bio-based, such as polyhydroxyalkanoate (PHA) and polylactic acid (PLA), and fossil-based, such as polybutylene succinate (PBS), poly(butylene adipate-*co*-terephthalate) (PBAT), and polycaprolactone (PCL). These bioplastics can be degraded by microbial enzymes or directly by microorganisms, helping with waste management and decreasing environmental pollution [15,16,17]. 

Polybutylene succinate (PBS) is a bioplastic synthesized by polycondensation between 1,4-butanediol and succinic acid [18]. Succinic acid can be obtained from renewable feedstock such as glucose, starch, or sucrose via fermentation by microorganisms [19]. To synthesize 1,4-butanediol, petroleum-based feedstocks are used [20]. It is an aliphatic polyester that has thermal and chemical resistance, flexibility, and melt processability. It has polyethylene (PE)- and polypropylene (PP)-like properties [21] and is widely used and discarded in everyday life. It can also be used for mulching films, compostable bags, and packaging [22], or can be mixed with other bioplastics to improve its properties. This makes PBS a promising material to replace synthetic plastics, preventing the accumulation of plastic waste. PBS can be degraded into water and carbon dioxide by hydrolytic or enzymatic degradation. In a previous study, when the hydrolytic method was applied, the weight loss of PBS was 31% [23]. Lipase produced by *Pseudomonas cepacia* can degrade PBS, resulting in 3.5% weight loss in 12 days [24]. However, the speed of PBS degradation is relatively slow, and using enzymes is not economically efficient. Therefore, we need to identify or engineer microorganisms that can degrade PBS directly. However, except for some fungi and mixed consortia species, we know little about other degrading microorganisms [25]. In this study, we screened for degrading strains and confirmed their biodegradation ability to expand the range of strains known and available to the scientific community. 

To identify a species of degrading bacteria, in this study, we screened PBS-degrading strains from marine samples and selected one with the best degradation activity using solid and liquid cultures at 30 °C, the temperature at which they were isolated. Additionally, we characterized the selected strain, *Terribacillus* sp. JY49, optimized the degradation conditions and observed the physical properties of the films (Table 1). Lastly, the degradability of other plastics was confirmed using clear-zone tests and esterase assay.

## 2. Materials and Methods

### 2.1. Chemicals 

All chemicals used in this study were of analytical grade. Chloroform, acetonitrile, poly-3-hydroxybutyrate-co-3-hydroxy-valerate(P(3HB-*co*-3HV)) pellets, *p*-nitrophenyl acetate, butyrate, octanoate, decanoate, and dodecanoate were obtained (Sigma-Aldrich, St. Louis, MO, USA). We obtained *p*-nitrophenyl hexanoate (Tokyo Chemical Industry, Tokyo, Japan). PHB pellets were obtained(Goodfellow Cambridge Ltd., Huntingdon, UK). PBAT and PBS pellets were obtained (Gio Soltech Co., Ltd. Wonju, Korea). Poly-3-hydroxybutyrate-co-4-hydroxybutyrate (P(3HB-co-4HB)) pellets were obtained (CJ, Suwon, Korea). Dichloromethane (DCM), fructose, and sucrose were obtained (Junsei Chemical Co., Tokyo, Japan). Glucose and xylose were obtained (Duksan Pure Chemicals, Ansan, Korea). Galactose was obtained (Daejung Chemicals, Siheung, Korea). 

### 2.2. Preparing Solid Media Containing Plastic 

For the preparation of media plates containing different plastics, 1 g of plastic pellets were dissolved in 40 mL DCM in a water bath at 60 °C. After adding 100 mL of distilled water, 2 mL of 2% Sarkosyl NL was added to the boundary of water and DCM [31,32]. The mixture was sonicated for 10 min with a 15 s pulse using Vibra Cell VCX500 (Sonics & Materials, Inc., Newtown, CT, USA). The amplitude was set at 30% to mix the contents uniformly. After sonication, the solvent was completely evaporated using a stirrer. Next, 1 g/L of plastic emulsion, uniformly dissolved in the aqueous phase of the solvent, was added to the marine broth (MB; Difco Laboratories, Detroit, MI, USA) containing peptone (5.0 g/L), yeast extract (1.0 g/L), ferric citrate (0.1 g/L), sodium chloride (19.45 g/L), magnesium chloride (5.9 g/L), magnesium sulfate (3.24 g/L), calcium chloride (1.8 g/L), potassium chloride (0.55 g/L), sodium bicarbonate (0.16 g/L), potassium bromide (0.08 g/L), strontium chloride (34.0 mg/L), boric acid (22.0 mg/L), sodium silicate (4.0 mg/L), sodium fluoride (2.4 mg/L), ammonium nitrate (1.6 mg/L), disodium phosphate (8.0 mg/L), and 2% of agarose. All the mixtures were then autoclaved for 15 min at 121 °C [33,34]. 

### 2.3. Screening Microorganisms for Their Ability to Degrade PBS

Soil samples were collected from different shores in Korea. These samples were diluted using autoclaved distilled water and spread on the MB agar plate containing 1 g/L of PBS. After incubation for 3–5 days at 30 °C, colonies that showed clear zones were isolated from MB-PBS agar plates. Each colony was inoculated in liquid MB media for 1 day, and stocks containing 20% (*w*/*v*) glycerol were stored at −80 °C for further use. Isolated colonies that produced clear zones were identified at the species level using 16s rRNA sequencing performed by Bionics (Seoul, Korea). Sequencing was conducted using the universal primer 27F. Partial sequences were aligned in the NCBI GeneBank database using BLASTn.

### 2.4. Characterizing the PBS-Degrading Strain

Antibiotic resistance (ampicillin, spectinomycin, gentamicin, kanamycin, and chloramphenicol) and hydrolase activity tests (amylase, protease, chitinase, and lipase) were conducted for the PBS-degrading strains. For the antibiotic resistance test, the strains were cultured on an MB agar plate containing 100 μg/mL ampicillin, 100 g/mL spectinomycin, 35 μg/mL chloramphenicol, 50 μg/mL kanamycin, and 25 μg/mL gentamicin. The appearance of colonies was considered a positive result [35].

Amylase activity was confirmed on starch agar plates containing 10 g/L soluble starch, 1 g/L yeast extract, and 15 g/L agar. After incubation, residual starch sources were stained using a 0.1 N iodine solution for 2 min. Protease activity was screened on skim milk agar plates containing 28 g/L skim milk, 5 g/L tryptone, 2.5 g/L yeast extract, 1 g/L glucose, and 15 g/L agar. The chitin agar plate was prepared by adding M9 medium, 1% soluble chitin, and 15 g/L agar and adjusting pH to 7. For lipase activity test, an agar plate containing 1% olive oil, 0.01% phenol red, 10 mM CaCl_2_, and 20 g/L agar was prepared, and the pH was adjusted to 7.3–7.4. All agar plates contained 2% salt to maintain salinity similar to that in the marine broth medium. In the case of lipase activity, only the formation of yellow zones was considered a positive result [35].

### 2.5. Solid and Liquid Culture to Monitor PBS Degradation

To characterize and optimize each microorganism, clear zone tests with various conditions were performed. To determine the optimal temperature to degrade PBS, each microbe was precultured in an optimal liquid medium for 24 h at 30 °C. Next, paper discs (Toyo Roshi Kaisha, Tokyo, Japan) were placed on the plate [36,37] and 10 μL of precultured cells were inoculated on the paper disc and incubated at 20 °C, 30 °C, 37 °C, and 42 °C for 7 days. We also inoculated the precultured cells on plates with 1% carbon source and 1%, 2%, 3%, and 4% of NaCl concentration and incubated them at 30 °C. The radius of clear zones was confirmed by measuring the distance between the paper disc and the endpoint of the clear zone. All experiments were performed in duplicates.

For liquid culture, PBS films were prepared using the conventional solvent-cast method [38]. We dissolved 0.2 g of PBS pellets in 100 mL of chloroform and heated the solution in a water bath at 60 °C until the pellets completely dissolved. The solvent containing dissolved PBS was kept in a fume hood until the solvent was completely evaporated and plastic films were formed. These films were cut into 40 mg pieces and sterilized with 70% ethanol and UV radiation on a clean bench. Prepared films were cultured in 100 mL flasks with 40 mL MB liquid medium. The liquid culture was performed under the same conditions as the solid culture. The time-dependent degradation rate was measured after 3, 5, 7, and 10 days of cultivation. We inoculated 2% JY49 and cultured it in a rotary shaker at 200 rpm. For further analysis, residual films were recovered, washed with distilled water several times, and freeze-dried for preparation of GC-MS samples. All experiments were performed in duplicate.

### 2.6. GC-MS Analysis

The amount of residual PBS and degradation yield were confirmed using GC-MS. To prepare GC-MS samples, the culture medium was centrifuged at 10,000× *g* for 10 min, and the residual PBS films were collected and washed several times with distilled water to remove residual medium components. Collected samples were lyophilized in Teflon-stoppered glass vials to completely remove water. For methanolysis of PBS, 1 mL of methanol/sulfuric acid (85:15 *v*/*v*) and 1 mL of chloroform were added to the vials and heated for 2 h at 100 °C. After 2 h, these vials were cooled down at room temperature, 1 mL of HPLC grade water was added to the vials, and the samples were vortexed for 1 min. The organic phase was extracted using a pipette and transferred to an e-tube containing anhydrous sodium sulfate to remove water. Samples were filtered (pore size, 0.2 μm) before injecting them into a GC-MS (Perkin Elmer, Waltham, MA, USA) equipped with a fused silica capillary column (Elite-5 ms, 30 m × 0.25 mm i.d. × 0.25 μm) and subjected to a linear temperature gradient for analysis (50 °C for 1 min, increased at 15 °C/min to 120 °C for 2 min and then increased at 10 °C/min to 300 °C for 10 min). The injector port temperature was 250 °C. Mass spectra were obtained using electron impact ionization at 70 eV, and scan spectra were obtained within the range of 45–450 *m*/*z* [39]. Selected ion monitoring was used for the detection and fragmentation analysis of the major products. To quantify the amount of residual PBS films, a calibration curve was obtained and degradation yield was calculated to indicate the degree of PBS degradation compared with the initial amount of PBS.

### 2.7. Analyzing the Physical Properties of PBS Films

To compare the change in the films’ surface after degradation, scanning electron microscopy (SEM) was used. To prepare the sample for SEM, degradation by *Terribacillus* sp. JY49 was performed for 0, 3, 5, 7, and 10 days, and residual PBS films were collected. Recovered films were washed with distilled water to remove the medium components and lyophilized. The films were coated with gold dust at 5 mA for 300 s, and back-scatter electron images were acquired using a TM4000Plus SEM instrument (Hitachi, Tokyo, Japan) at 5 kV [38]. To observe the films at high magnification, SU-8010 FE-SEM (Hitachi, Tokyo, Japan) was used at 5 kV.

To determine the molecular weight changes of the PBS films during degradation, we used gel permeation chromatography (YOUNG IN Chromass, Anyang, Republic of Korea). To prepare the samples, PBS films were recovered after degradation, dissolved in 1 mL of chloroform, and heated on a heat block at 60 °C. This solution was filtered through a syringe filter (pore size, 0.2 μm; Chromdisc, Daegu, Korea). A high-performance liquid chromatography (HPLC) apparatus was used for the analysis, consisting of a loop injector (Rheodyne 7725i), an isocratic pump with dual heads (YL9112), column oven (YL9131), columns (Shodex, K-805, 8.0 mm I.D. × 300 mm; Shodex, K-804, 8.0 mm I.D. × 300 mm), and an RI detector (YL9170). We injected 60 μL of the sample without air bubbles. Chloroform was used as the mobile phase, and the flow rate was maintained at 1.0 mL/min at 35 °C. The data were analyzed using YL-Clarity software for a single YL HPLC instrument (YOUNG IN Chromass). The molecular weight was analyzed in relation to polystyrene standards ranging from 5000 to 2,000,000 g/mol [32].

The changes in functional groups in the PBS films were observed using Fourier-transform infrared spectroscopy (Nicolet 6700, Thermo Fisher Scientific, Waltham, MA, USA). We recorded 32 scans for each spectrum using an auto base in the scanning range of 4000 to 600 cm^−1^ and a resolution of 4 cm^−1^.

### 2.8. Confirming the PBS Monomer Effect

Succinic acid and 1,4-butanediol were added to MB medium and cultured with *Terribacillus* sp. JY49 to confirm the effect of PBS monomers. This culture was incubated at 30 °C and 200 rpm by adding 10 mM of each monomer. The pH range was adjusted to 7.6 ± 0.2. We sampled 300 µL of culture medium after 24 h and 48 h to measure the optical density. After checking the optical density of each sample, the remaining culture medium was centrifuged at 13,000 rpm for 10 min to obtain the supernatant to check the monomer consumption using high-performance liquid chromatography (HPLC). Supernatants were diluted 10-fold with HPLC water and filtered (pore size, 0.2 μm). The HPLC (Perkin Elmer, Waltham, MA, USA) was equipped with a refractive index detector and a UV-vis detector. PBS monomers were separated on an Aminex HPX-87H column (300 × 7.8 mm internal diameter) (Bio-Rad, Hercules, CA, USA). The flow rate of the mobile phase was maintained at 0.6 mL/min using 0.004 mol/L sulfuric acid (H_2_SO_4_). The oven temperature was set to 60 °C during the operation [40].

We also confirmed the minimal inhibitory concentration (MIC) to check the monomers’ effect on the growth of *Terribacillus* sp. JY49. Monomer solution at a concentration of 128 mM was prepared. In a 96-well plate, monomer concentration ranging from 0.25–64 mM was prepared, and 2% of strain was inoculated. After incubating for 24 h, optical density was measured to check the minimal inhibitory concentration of PBS monomers.

### 2.9. Esterase Activity Assay with p-Nitrophenyl Esters

To check the esterase activity of the degrading strains, six *p*-nitrophenyl esters were used as substrates: *p*-nitrophenyl acetate, *p*-nitrophenyl butyrate, *p*-nitrophenyl hexanoate, *p*-nitrophenyl octanoate, *p*-nitrophenyl decanoate, and *p*-nitrophenyl dodecanoate. The enzyme reactions comprised 180 μL of 50 mM phosphate buffer (pH 7.4), 10 μL of supernatant (collected from the liquid culture centrifuged at 4 °C and 13,000 rpm for 10 min), 5 μL of substrates dissolved in acetonitrile, and 5 μL of ethanol. These mixtures were reacted in an incubator at 37 °C for 10 min. Absorbance was measured at 405 nm in a 96-well plate to confirm esterase activity [41].

## 3. Results

### 3.1. Screening Polybutylene Succinate (PBS)-Degrading Strains from Marine Samples

**Figure 1:**
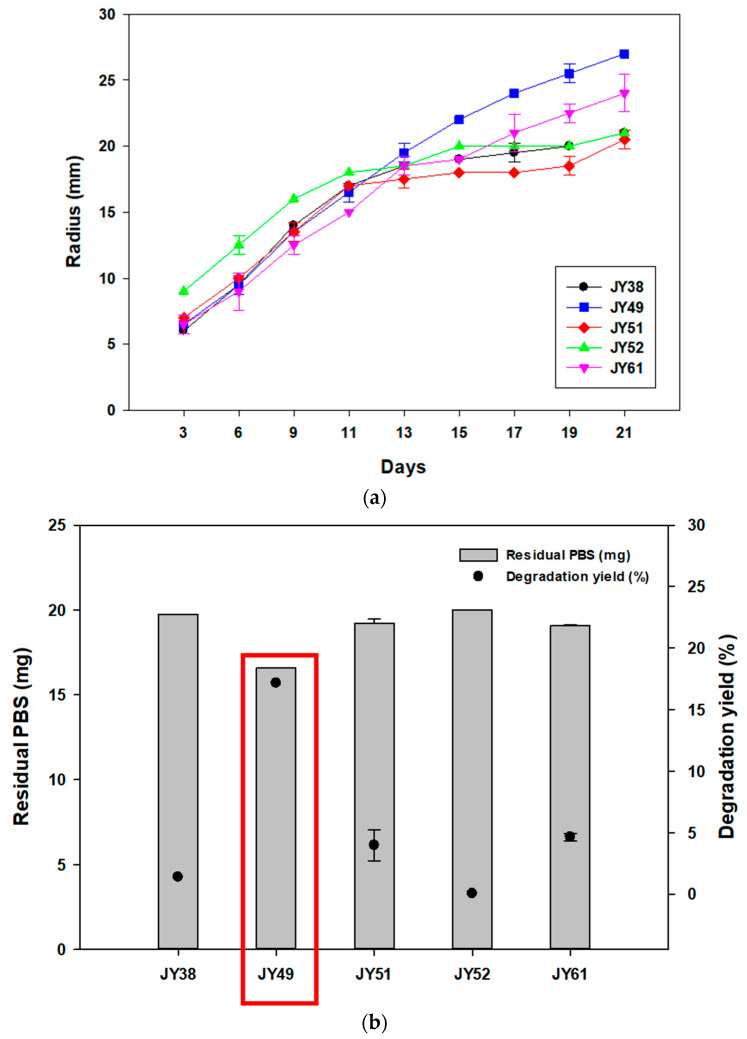
Comparing PBS-degrading strains using solid and liquid cultures and identifying the selected strains. (**a**) The changes in radius size of clear zones for 21 days at 30 °C. (**b**) Amount of residual PBS and degradation yield of five PBS-degrading strains using GC-MS analysis.

To screen the strains with the ability to degrade PBS, we collected various marine samples. These samples were spread on marine broth-containing agar plates containing 1% PBS emulsion, and strains forming clear zones were selected. Five different microbes were screened from these samples (Table S1), and all strains were isolated at 30 °C. To select one strain with the best degradation activity towards PBS, the radius of clear zones was compared, and the residual amount of PBS was analyzed using GC-MS. In the case of liquid culture, 20mg of PBS films were added to the media. These experiments were conducted at 30 °C, the temperature at which the strains were isolated. In clear-zone tests, JY52 had the largest radius until 11 days, but JY49 showed larger and more transparent clear zones after 21 days (Figure 1a). Comparing GC-MS results of the two, the degradation yield of JY49 was higher than that of other strains (Figure 1b), and it was selected for subsequent experiments.

**Figure 2:**
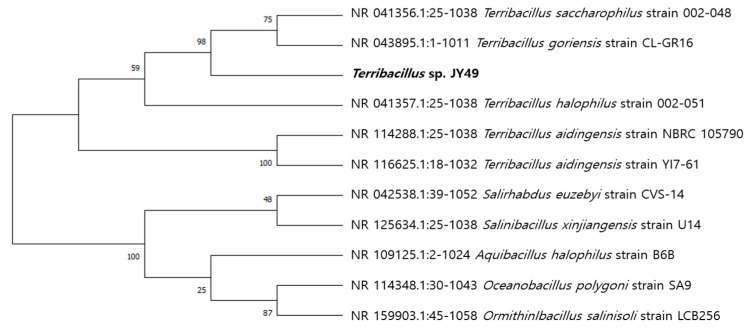
Phylogenetic tree of *Terribacillus* sp. JY49 according to 16S rRNA sequencing.

We found JY49 to have the highest similarity with *Terribacillus gorinesis* (99.51%) using 16S rRNA sequencing (Figure 2). *Terribacillus* sp. are gram-positive strains that can live in aerobic and halophilic conditions. To characterize this strain, hydrolysis activity and antibiotic resistance tests were performed. Except for protease activity, activity was confirmed in other hydrolysis tests, and the strain was not resistant to any antibiotics (Table S2). Several types of *Terribacillus* sp. strains have been published in previous studies (Table 2). However, there have been few reports of producers or degraders of substances. Therefore, this study is the first report of PBS-degrading *Terribacillus* species.

### 3.2. Optimizing Temperature Conditions for PBS Degradation by JY49

**Figure 3:**
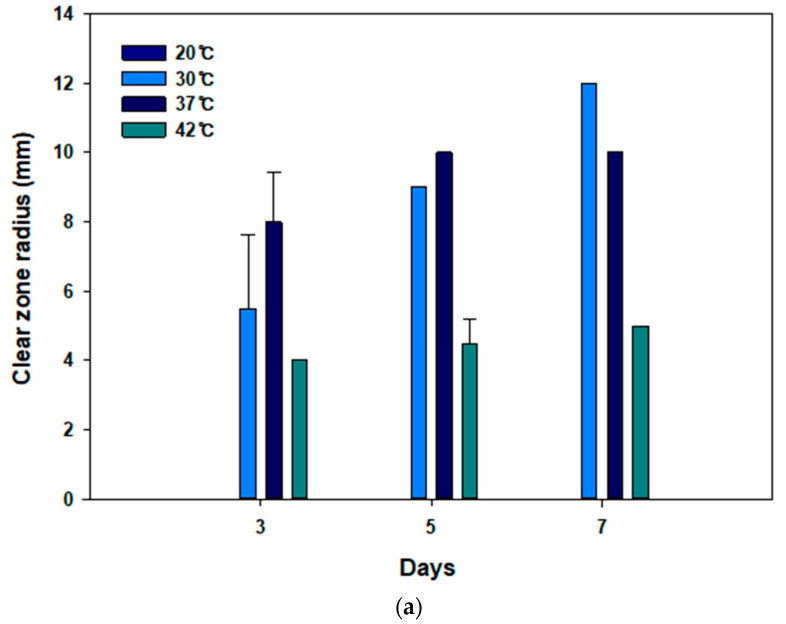

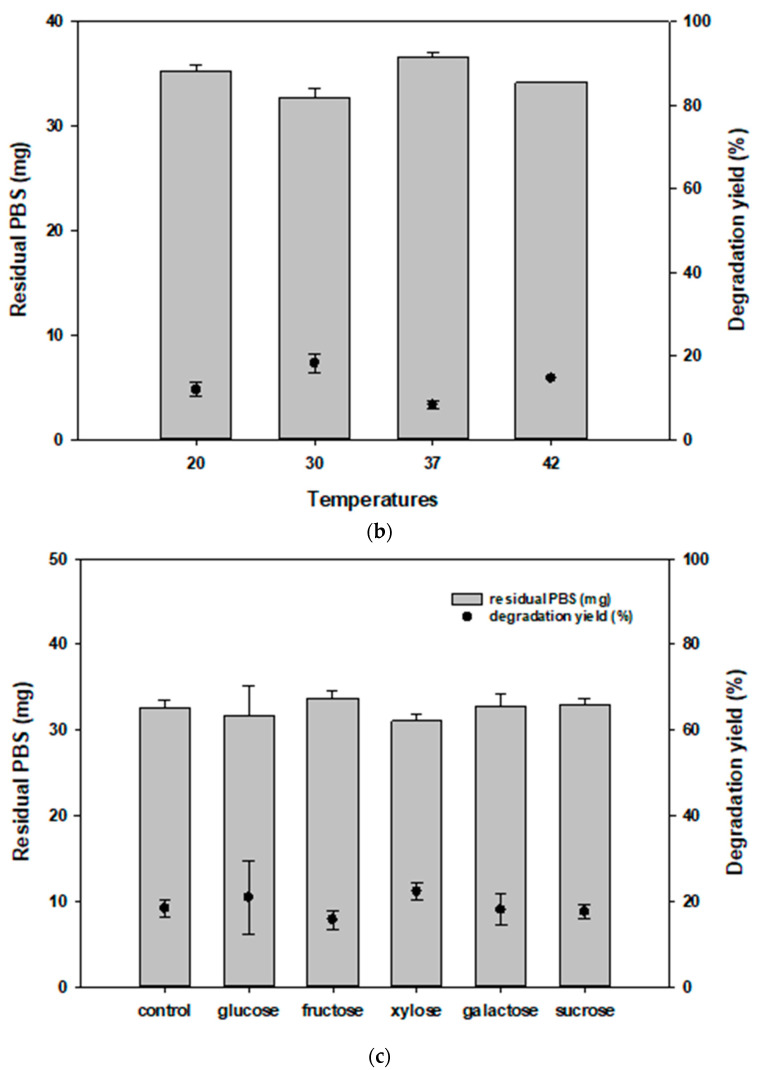

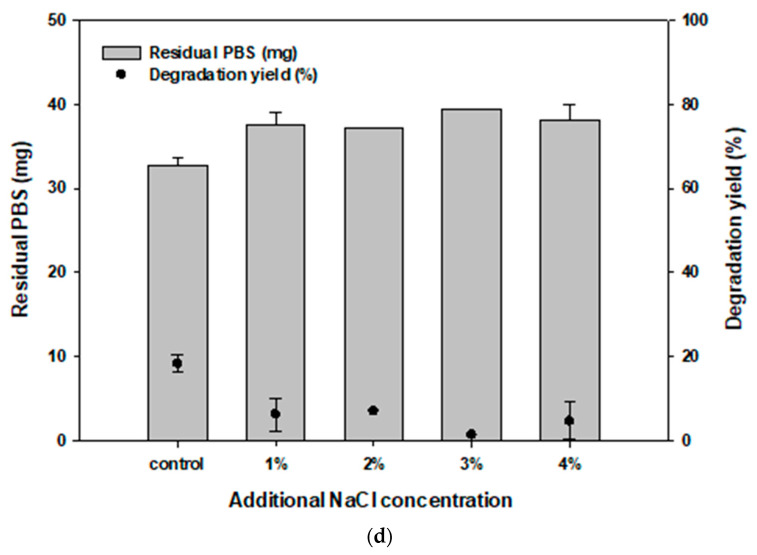
Optimizing the conditions for degrading PBS. (**a**) The changes in clear zone size at different temperatures (20 °C, 30 °C, 37 °C, 42 °C). (**b**) The results of GC-MS analysis to compare the amounts of residual PBS and degradation yield according to temperatures. (**c**) Five different carbon sources (glucose, fructose, xylose, galactose, and sucrose) were used to compare their effect on degradation at 30 °C using GC-MS. (**d**) Comparison of degradation yield according to salt concentration at 30 °C.

*Terribacillus* sp. JY49 was isolated from MB medium at 30 °C. Reportedly, the optimal growth temperature of this strain is 30 °C [46]. To test the best temperature to degrade PBS, clear-zone tests and liquid culture were conducted at various temperatures (20 °C, 30 °C, 37 °C, and 42 °C) for 7 days. At 37 °C, clear zones formed rapidly in the beginning, but the largest and most transparent clear zone appeared at 30 °C after 7 days. Moreover, *Terribacillus* sp. JY49 had the ability to degrade PBS at 42 °C, but the size of the clear zone was small and did not increase rapidly compared with those at 30 °C and 37 °C. However, a clear zone was not visible at 20 °C (Figure 3a). After liquid culture, a residual amount of PBS films was measured using GC-MS. *Terribacillus* sp. JY49 can degrade PBS at all temperatures. At 30 °C, degradation yield was the highest (Figure 3b). We expect that the optimal growth temperature affects PBS degradation.

### 3.3. Effect of Carbon and Salt Concentration on PBS Degradation

To confirm the effect of carbon sources on PBS degradation, five carbon sources (glucose, fructose, galactose, xylose, and sucrose) were selected. We compared the size of clear zones on the MB agar media containing 1% of carbon sources and PBS emulsion at 30 °C. After 7 days, clear zones were observed for all carbon sources except sucrose. The radius size was similar to that in the control in the case of xylose and glucose. Plates containing fructose also showed clear zones, but with poor transparency compared with other carbon sources. GC-MS results showed that xylose-containing media had a degradation yield of 22.3%, which was higher than the 18.8% in the control (Figure 3c). The degradation yield was 20.8% for glucose. However, degradation yield decreased compared with that in the control when fructose, galactose, and sucrose were added to the medium. These carbon sources are expected to affect the growth of strain and enzyme production involved in degradation, and therefore, it appears that different degradation yield depending on carbon sources are shown in the above results.

Clear zone size and residual amount of PBS were evaluated by adding NaCl to the solid and liquid medium. When 1% of NaCl was added, this strain showed a larger clear zone radius compared with the control on solid medium. At other salt concentrations, clear zones were observed, but the transparency and size were weaker than that for 1% NaCl. In liquid culture, degradation yield decreased after adding NaCl to the medium (Figure 3d). Compared with the control, when NaCl was added to 1% and 2% to liquid medium, the degradation yield was 6.2% and 7.0%. In the presence of NaCl, PBS could be fragmented by JY49, but the amount of residual film did not change significantly. *Terribacillus* sp. is moderately halotolerant [46]. Therefore, in the presence of excess NaCl, degradation can occur even though degradation yield was not high compared with control conditions.

### 3.4. Time-Dependent Monitoring of PBS Degradation

**Figure 4:**
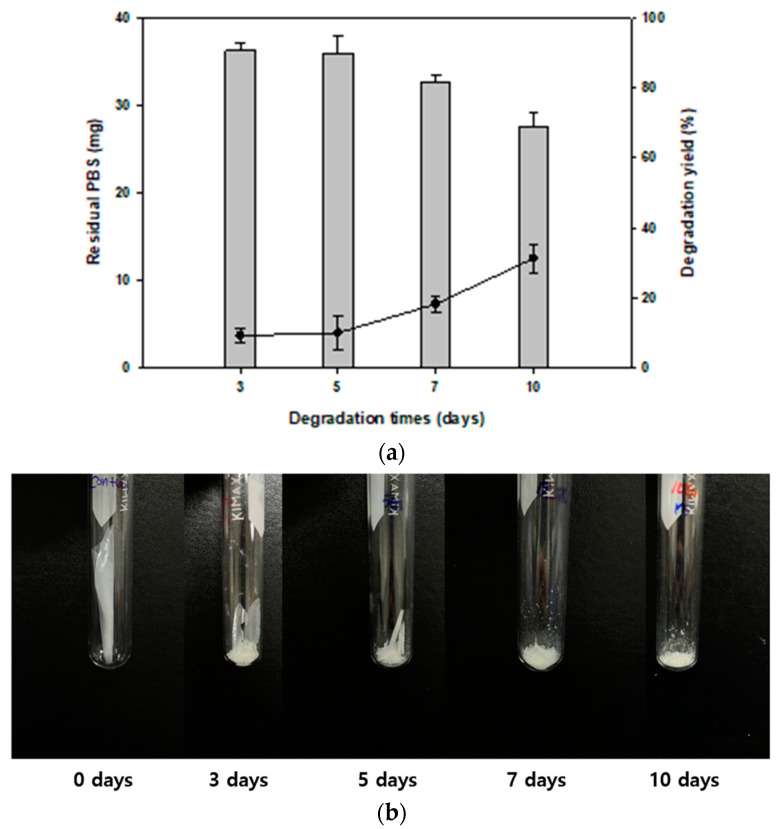
Confirming the degree of PBS degradation based on cultivation period (**a**) Residual amount of PBS and degradation yield was confirmed using GC-MS. (**b**) Recovered PBS films after lyophilization. The amount of PBS films decreased, and the films fragmented with time.

We analyzed the degradation pattern in liquid culture at different time intervals. This experiment was conducted in MB liquid medium at 30 °C with 40 mg of PBS films. The results showed that the film was gradually degraded, and after 10 days, the residual amount of PBS was 27.5 mg and the degradation yield was 31.4% (Figure 4a). Compared with control films, the shape of the films changed, and they gradually fragmented (Figure 4b). However, after 10 days, PBS film degradation yield did not increase (data not shown). This might be due to the metabolites produced from degrading PBS (such as PBS monomers) that might inhibit the growth of *Terribacillus* sp. JY49, or the production of enzymes associated with degradation. In previous studies, several systems were screened for degrading PBS, and they showed high weight loss in liquid and soil conditions. However, it was confirmed that most of the degradation strains were fungi and a mixed consortium of bacteria. Therefore, *Terribacillus* sp. JY49 is an important PBS-degrading species.

### 3.5. Changes in Physical Properties of PBS Films

**Figure 5:**
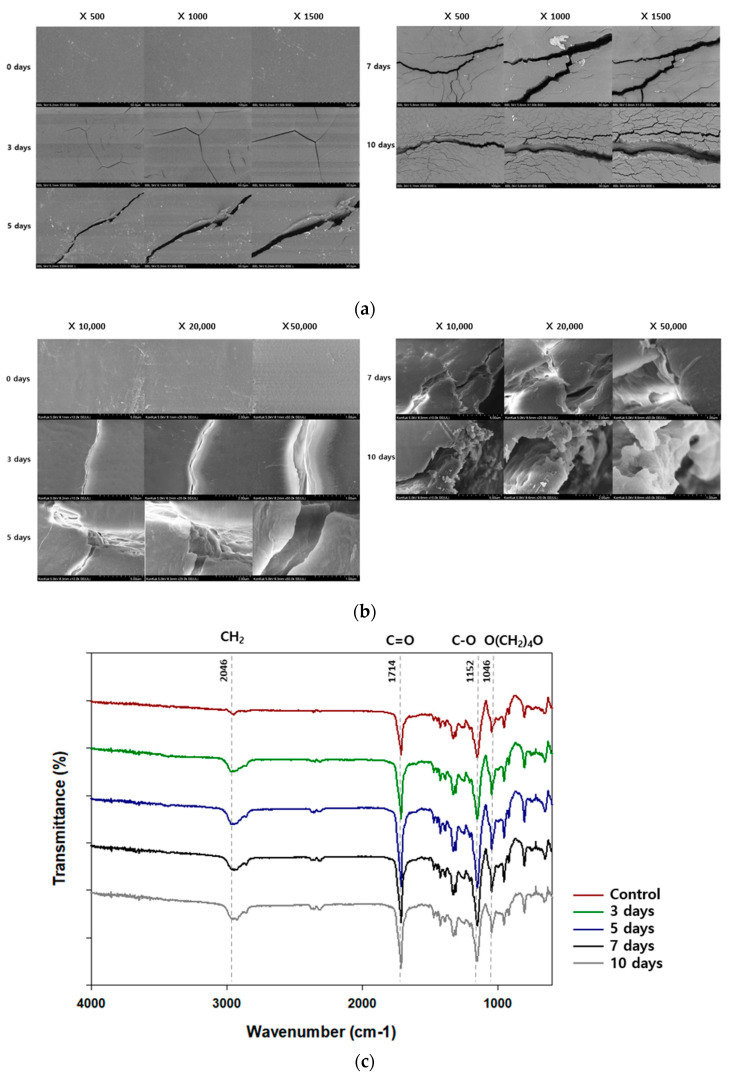
Changes in the surface and functional group of PBS films after degradation. (**a**) The surface of PBS was observed using SEM after 3, 5, 7, and 10 days of cultivation. (**b**) Cracks and rough surfaces formed after degradation were also observed using FE-SEM with high magnification. (**c**) Changes in functional groups were confirmed using FI-IR analysis. After degradation by *Terribacillus* sp. JY49, peak intensities changed with time.

As PBS degrades, the physical properties of films may be affected, such as the surface, molecular weight, and functional groups. Changes in the surface of PBS films were observed using SEM and FE-SEM with high magnification PBS film surface before degradation was smooth without any cracks and scratches (Figure 5a). At the beginning of the degradation, cracks began to appear, and as the degradation progressed, the number and depth of the cracks increased. After 10 days, PBS films with large cracks and rough surface were observed. FE-SEM showed similar results. Surface changes in the films demonstrate that *Terribacillus* sp. JY49 can degrade PBS (Figure 5b).

Additionally, molecular weight was measured using gel permeation chromatography during the degradation (Table 3). Number average molecular weight (M_n_), weight average molecular weight (M_w_), and polydispersity index (PDI) of PBS films were determined. Before degradation, the values of M_w_ and M_n_ were 3.87 × 10^4^ and 9.49 × 10^4^, respectively. As the degradation progressed, the M_n_ and M_w_ values decreased because the molecular chain was degraded by *Terribacillus* sp. JY49. After 10 days, films that were recovered from culture medium had M_n_ of 2.88 × 10^4^ and M_w_ of 6.82 × 10^4^. However, PDI values did not show significant changes like in the case of PHA and PBAT degradation [32,40]. Usually, PBS is degraded because enzymes produced from microorganisms attack the ester bond in the main chain of PBS. Lipase and cutinase, which are esterases, can attack and degrade PBS. The esterase-based enzyme produced by *Terribacillus* sp. JY49 might cut the end of polymers. However, it did not cut the inside of the polymer and hence, did not affect the molecular weight distribution.

PBS films were analyzed after degradation using Fourier transform infrared spectroscopy to check the change in functional groups (Figure 5c). *Terribacillus* sp. JY49 was cultured with PBS films for 10 days, and the residual PBS films were recovered. After PBS degradation, the peak intensity at 2945 cm^−1^ had changed. This peak indicates a CH_2_ stretching bond. The change of this peak intensity indicates a variation in alkane groups. A change of peak intensity at 1714 cm^−1^ and 1152 cm^−1^, corresponding to a C=O stretching bond and C–O bond, could be confirmed. These bonds are a part of the ester bond. Because PBS has ester bonds, the changes in the two peaks indicate that PBS was degraded by this strain. In addition, the peak in the range of 1046 cm^−1^ indicates the O(CH_2_)_4_O vibration. This functional group exists in PBS structures, therefore, degradation by *Terribacillus* sp. JY49 can change the chemical structure of PBS.

### 3.6. Effect of PBS Monomers such as Succinic Acid and 1,4-Butanediol on the Growth of JY49

PBS is composed of succinate and 1,4-butanediol monomers. Hence, when JY49 biodegrades PBS, these monomers are released and can affect the growth of this strain. Therefore, we confirmed monomer consumption and growth of JY49 through LC analysis and O.D. measurement, respectively, in a pH-controlled environment. Compared with control, the residual amount of succinic acid was decreased and completely consumed after 48 h (Figure S1a). 1,4-butanediol consumption was confirmed by its decreasing concentration with time. Increased growth was also observed after adding these two monomers (Figure S1b). This means that monomers of PBS were not significantly inhibitory factors in an environment with adjusted pH.

We also checked minimal inhibitory concentration (MIC) of the monomers. When the concentration of succinic acid reached ≥8 mM, the O.D value decreased sharply, indicating that high concentration of succinic acid inhibited the growth of JY49 (Figure S2a). However, the concentration of 1,4-butanediol was not significantly related to the growth of JY49 (Figure S2b). Therefore, as degradation continues, the released monomers may affect the growth of JY49, which is also expected to affect the degradation yield. This may be one of the reasons for the stalled degradation of PBS.

### 3.7. Degradability of Other Bioplastics by Terribacillus sp. JY49

**Figure 6:**
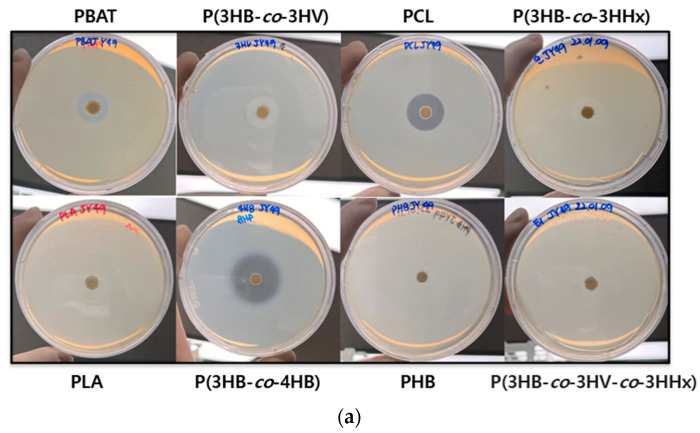

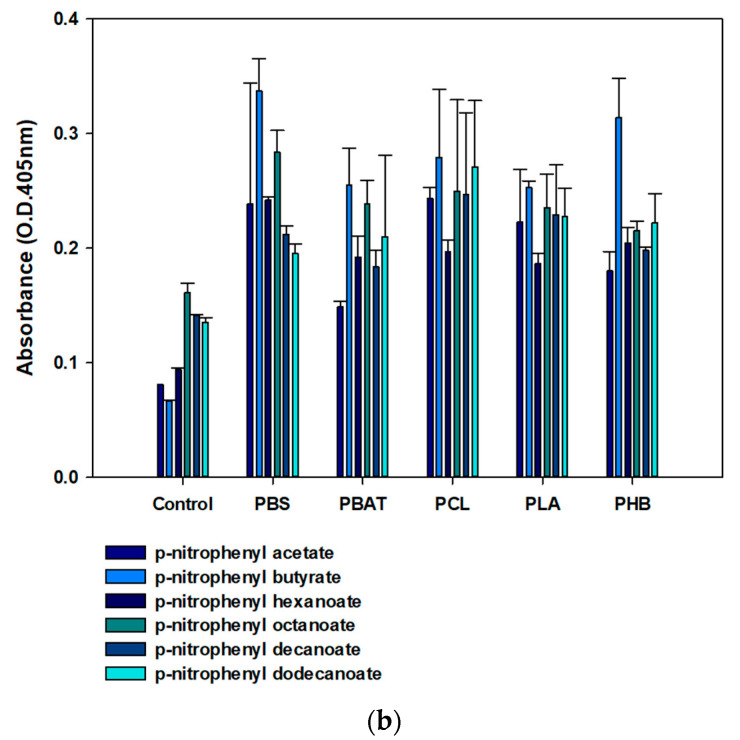
Degradation ability of JY49 on other bioplastics. (**a**) Formation of clear zone was tested on plates containing other plastics at 30 °C for 7 days. (**b**) Esterase assay was conducted with various p-nitrophenyl esters, and absorbance was measured at 405 nm.

To test the degradability of other plastics by *Terribacillus* sp. JY49, a clear zone test was performed. Experiments were performed using an MB agar medium containing different plastics at 30 °C. This strain showed clear zones on PBAT, PCL, P(3HB-*co*-3HV), and P(3HV-*co*-4HB)-containing plates (Figure 6a), which indicates that *Terribacillus* sp. JY49 can degrade other bioplastics. Among them, the plate containing P(3HV-*co*-4HB) showed the largest and most transparent clear zone. However, there were no formations of clear zones on PLA, PHB, P(3HB-*co*-3HV-*co*-3HHx). The possibility of different plastic degradability of *Terribacillus* sp. JY49 was confirmed through clear zone testing.

To check the esterase activity that could degrade other bioplastics and monitor whether each enzyme produced with different bioplastics has a different activity towards substrates of different lengths, different kinds of substrates were selected (*p*-nitrophenyl acetate, *p*-nitrophenyl-butyrate, *p*-nitrophenyl hexanoate, *p*-nitrophenyl octanoate, *p*-nitrophenyl decanoate, and *p*-nitrophenyl dodecanoate) [41,47]. Enzyme reaction was performed on 96-well plates for 10 min, and the absorbance was measured at 405 nm. After reaction, the color changes to yellow in the wells due to the extracellular esterase enzyme activity from *Terribacillus* sp. JY49. This strain showed little difference in esterase activity with respect to different bioplastics (Figure 6b), and any small change could be due to the many different enzymes such as protease and cutinase degrading the different bioplastics.

## 4. Conclusions

The use of bioplastics is drawing attention as a solution to alleviate environmental problems caused by plastic waste. Among them, PBS is a promising replacement for synthetic plastics derived from petroleum. Because the mechanism of PBS degradation is still unknown and the number of degrading strains is small, we aim to find PBS-degrading bacterial strains and confirmed the biodegradation activity. In this study, we screened *Terribacillus* sp. JY49, which has the highest similarity with *Terribacillus goriensis*, and which is the best PBS degrader. It has optimum degradation activity at 30 °C in solid and liquid cultures, and the degradation yield changes with carbon sources and salt concentration. We analyzed the roughness and erosions on the surface of the films through SEM, the reduction in molecular weight using gel permeation chromatography, and change in functional groups via FT-IR. This strain also has esterase activity—confirmed by measuring the absorbance—which can aid the degradation of PBS. Additionally, it showed degradability for other bioplastics such as PBAT, PCL P(3HB-*co*-3HV), and P(3HV-*co*-4HB). Therefore, *Terribacillus* sp. JY49 can be a useful and promising bacterial strain that can be applied for PBS degradation.

## Figures and Tables

**Table 1:** List of PBS-degrading strains reported in previous studies.

Strain	Type	Temp	Period (Days)	Weight Loss (%)	Biodegradation Percentage (%)	Condition	Reference
*Fusarium* sp. FS1301	Fungi	30 °C	21	80%	-	Liquid	[26]
*Bionectria ochroleuca* BFM-X1	Fungi	30 °C	30	60%	-	Soil	[27]
*Aspergillus fumigatus*	Fungi	30 °C	30	80%	-	Soil	[28]
*Fusarium solani*	Fungi	-	14	-	2.8%	Soil	[29]
*Aspergillus versicolor*, *Penicillium*, *Bacillus*, *Thermopolyarpora*	Consortia	-	90	-	71.9% (powder) 60.7% (film) 14.1% (granule)	Soil	[30]
*Terribacillus goriensis*	Bacteria	30 °C	10	-	31.4%	Liquid	This paper

**Table 2:** List of previously reported *Terribacillus* sp.

Strains	Strain No.	Remark	Isolated Site	Reference
*Terribacillus saccharophilus*	KCTC 13936, DSM 21620	Characterization *	Field soil	[42]
*Terribacillus halophilus*	KCTC 13936, DSM 21620	Characterization *, Antimicrobial behavior **	Field soil	[42,43]
*Terribacillus aidingensis*	DSM 28352, CGMCC 1.8913	Characterization *	Soil from lake	[44]
*Terribacillus goriensis*	KCCM 42329, DSM 18252	Characterization *	Surface of sea water	[45]

* Characterization: report of screened and novel strains with basic experiments; ** Antimicrobial behavior: report of antimicrobial activity of strains.

**Table 3:** Change in molecular weight of PBS films, analyzed using gel permeation chromatography.

Day	M_n_ × 10^4^	M_w_ × 10^4^	PDI
0	3.87	9.49	2.45
3	3.57	8.56	2.40
5	3.38	8.31	2.46
7	3.11	7.63	2.45
10	2.88	6.82	2.37

## Data Availability

Not applicable. No new data were created or analyzed in this study. Data sharing is not applicable to this article.

## Supplementary Materials

The following supporting information can be downloaded at: https://www.mdpi.com/article/10.3390/polym14193978/s1. Table S1: PBS-degrading strains isolated from marine samples and identified via 16s rRNA sequencing, Table S2: Characterization of *Terribacillus* sp. JY49 using hydrolysis and antibiotic resistance test, Figure S1. Confirmation of monomer consumption and growth of *Terribacillus* sp. JY49, Figure S2: Minimal inhibitory concentration (MIC) of the monomers were checked.
