# Supplementary material for: Acceleration of Polybutylene Succinate Biodegradation by Terribacillus sp. JY49 Isolated from a Marine Environment
Source: Polymers (Basel). 2022 Sep 23;14(19):3978. doi: 10.3390/polym14193978 (PMC9571400; doi:10.3390/polym14193978)
Supplement: Supplementary file 1 [file polymers-14-03978-s001.zip › polymers-1904283-supplementary.pdf]

polymers

MDPI

Supplementary Material

Acceleration of polybutylene succinate biodegradation by *Terribacillus* sp. JY49 isolated from a marine environment

Su Hyun Kim [1], Jang Yeon Cho [1] Do Hyun Cho [1], Hee Ju Jung [1], Byung Chan Kim [1], Shashi Kant Bhatia [1,2], See-Hyoung Park [3], Kyungmoon Park [3] and Yung-Hun Yang [1,2,*]

1 Department of Biological Engineering, College of Engineering, Konkuk University, Seoul 05029, Korea
2 Institute for Ubiquitous Information Technology and Applications, Konkuk University, Seoul 05029, Korea
3 Department of Biological and Chemical Engineering, Hongik University, Sejong 30016, Korea
* Correspondence: seokor@konkuk.ac.kr; Tel.: +82-2-450-2-3936

Table S1. PBS-degrading strains isolated from marine samples and identified via 16s rRNA sequencing.

Isolates	Related strains	Identity	Query	Isolation temperature
JY38	*Bacillus subtilis*	97.32%	99%	30 ℃
JY49	*Terribacillus goriensis*	99.51%	97%	30 ℃
JY51	*Bacillus subtilis* strain IAM 12118	99.63%	99%	30 ℃
JY52	*Bacillus subtilis* strain DSM 10	98.24%	81%	30 ℃
JY61	*Bacillus zhangzhouensis*	99.52%	98%	30 ℃

Table S2. Characterization of *Terribacillus* sp. JY49 using hydrolysis and antibiotic resistance test.

Hydrolysis test		Antibiotic resistance test	
Amylase	-	Ampicillin	-
Protease	++	Gentamycin	-
Chitinase (shrimp shell)	-	Spectinomycin	-
Lipase (olive oil)	-	Chloramphenicol	-
		Kanamycin	-

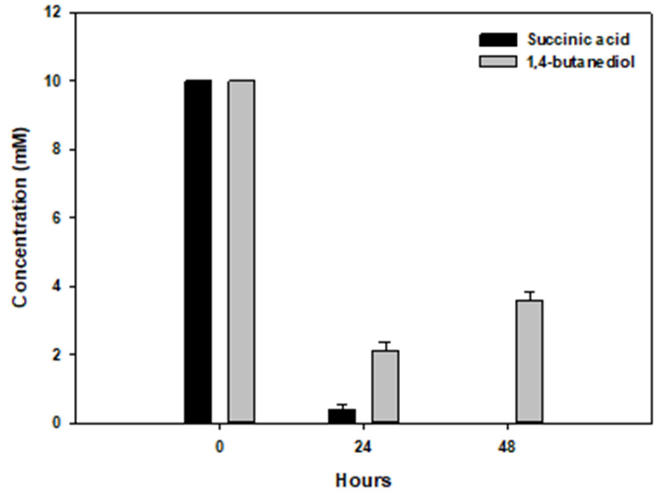

(a)

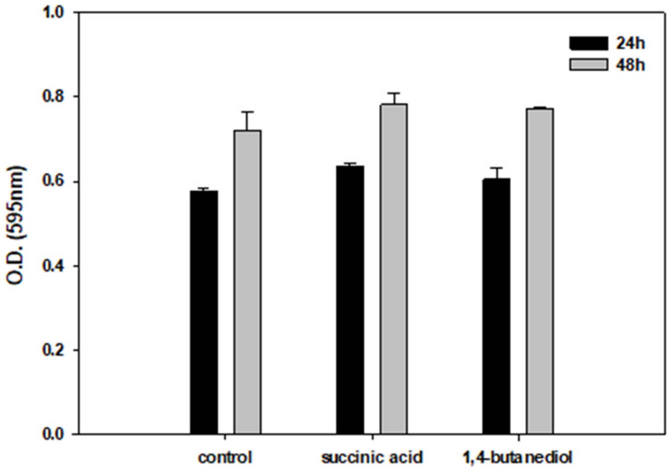

(b)

Figure S1. Confirmation of monomer consumption and growth of *Terribacillus* sp. JY49 (a) Consumption of monomer-were tested via LC analysis. Residual amount of succinic acid was decreased and completely consumed after 48h. Also, 1,4 butanediol consumption was confirmed. (b) Growth of JY49 was increased after adding these two monomers. .

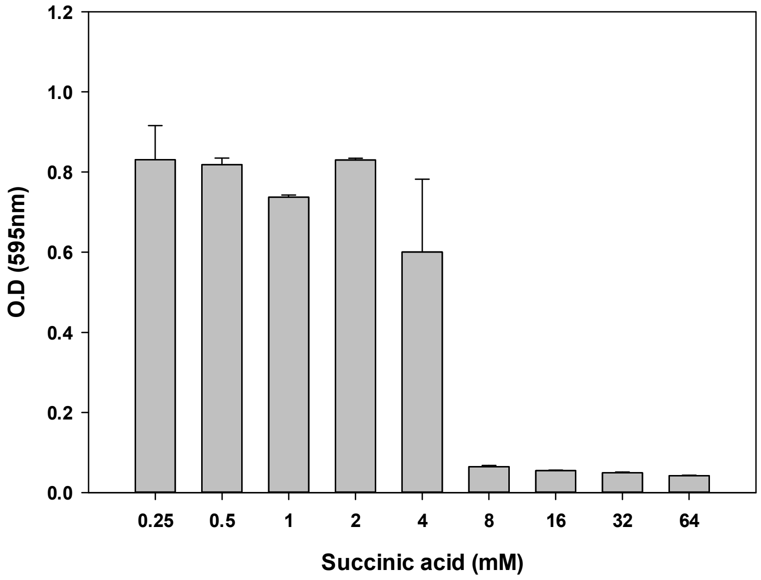

(a)

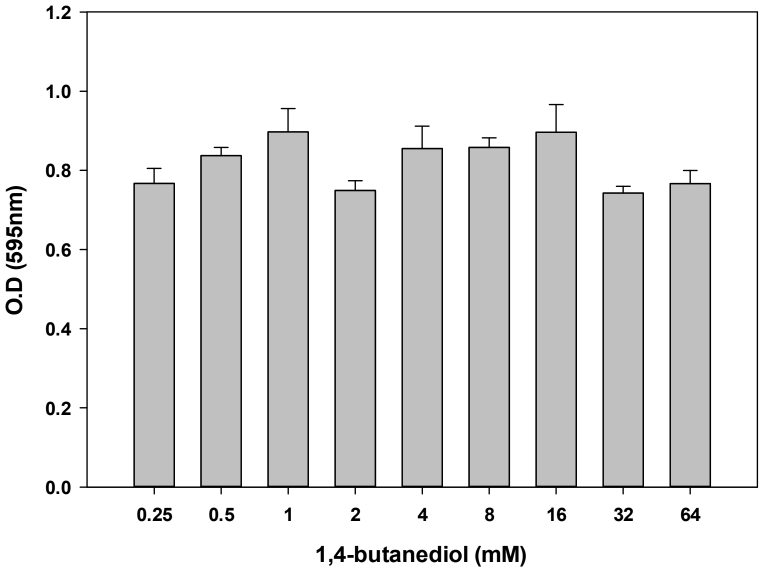

(b)

Figure S2. Minimal inhibitory concentration (MIC) of the monomers were checked. (a) When the concentration of succinic acid was more than 8Mm, the O.D value decreased sharply. (b) the concentration of 1,4-butanediol was not significantly related to the growth of *Terribacillus* sp. JY49.
